# Case Report: Ziprasidone induced neuroleptic malignant syndrome

**DOI:** 10.12688/f1000research.51094.1

**Published:** 2021-02-17

**Authors:** Yub Raj Sedhai, Alok Atreya, Prabin Phuyal, Soney Basnyat, Sagar Pokhrel

**Affiliations:** 1 Department of Internal Medicine, Virginia Commonwealth University School of Medicine, South Hill, VA, USA; 2 Lumbini Medical College, Lumbini, Palpa, 32500, Nepal; 3 Department of Internal Medicine, BP Koirala Institute of Health Sciences, Dharan, Nepal; 4 Division of Geriatric Medicine, Department of Internal Medicine, University of Michigan, Ann Arbor, MI, USA; 5 Department of Critical Care Medicine, Institute of Medicine, Maharajgunj Medical Campus, Kathmandu, Bagmati, Nepal

**Keywords:** Neuroleptic Malignant Syndrome (NMS), Neurologic emergency, Parkinson’s disease, Ziprasidone

## Abstract

Neuroleptic malignant syndrome (NMS) is a well-recognized neurologic emergency. It presents with classic features including hyperthermia, autonomic instability, muscle hypertonia, and mental status changes. The syndrome is potentially fatal and is associated with significant morbidity due to complications such as rhabdomyolysis, acute kidney injury, and ventricular arrhythmias due to the trans-cellular electrolyte shift. NMS is conventionally associated with the first-generation antipsychotic agents, however, has been described with the use of atypical and novel antipsychotics including Ziprasidone. A case of NMS with Ziprasidone use at the therapeutic dose is reported here.

## Introduction

Neuroleptic malignant syndrome (NMS) is a well-recognized neurologic emergency associated with antipsychotic (neuroleptic) drugs. NMS is characterized by hyperthermia, autonomic instability, severe muscle hypertonia, and mental status changes ^1^. NMS has been conventionally associated with typical antipsychotics like Haloperidol and Fluphenazine ^1^. However, recent studies have shown that every class of antipsychotic can cause NMS including low potency agents like Chlorpromazine, and second-generation antipsychotics like Clozapine, Risperidone, and Olanzapine, and antiemetics like Metoclopramide and Promethazine ^2, 3^. NMS results in muscle breakdown, leading to potential sequelae of rhabdomyolysis and acute kidney injury ^4^. Muscle breakdown results in a large transcellular shift which can result in ventricular arrhythmias and can be potentially fatal with a reported mortality of 10–20% ^4^. NMS can result from the use of high-potency or depot formulations of antipsychotics, however, clinicians should be well aware that the use of parenteral antipsychotics for sedation and control of agitation is an important cause of NMS in the inpatient setting ^5^. Intravascular volume depletion and dyselectrolytemia are important risk factors of NMS ^5^. Herewith we present a case of an elderly male, with a history of parkinsonism, who was admitted with toxic metabolic encephalopathy related to urinary tract infection (UTI). He developed NMS after administration of Ziprasidone for control agitation and combative behavior.

## Case presentation

A 78-year-old male was admitted for urinary tract infection complicated with toxic metabolic encephalopathy. He had past medical history of parkinsonism along with three vessel coronary artery bypass graft (CABG). He was on prescription medications including levodopa/carbidopa. On the night of hospitalization, he developed agitation and combative behavior which was treated with several doses of intramuscular Ziprasidone. He received a total of 30 mg intramuscular Ziprasidone over 12 hours. The following morning the on-duty nurse noted the patient to have generalized muscular rigidity. Assessment of vital signs revealed the temperature of 107° F, heart rate of 120 beats per minute, blood pressure 160/90 mmHg, with oxygen saturation of 94% on room air. A rapid response alert was called.

On further evaluation, he had generalized muscular rigidity, involving both flexor and extensor compartments in the bilateral upper and lower extremity, muscles of the abdominal wall, back, neck, and jaw suggestive of generalized hypertonia. He had diaphoresis with hyperthermia. Neurologic examination, revealed unresponsiveness, with no eye-opening in response to pain, no verbal response, and no motor response accounting for a Glasgow coma scale of three. Examination of the cardiovascular system revealed sinus tachycardia and was otherwise unremarkable. Chest auscultation revealed minimal mid-inspiratory crackles at the base. Systemic examination was otherwise unremarkable.

**Figure 1.**
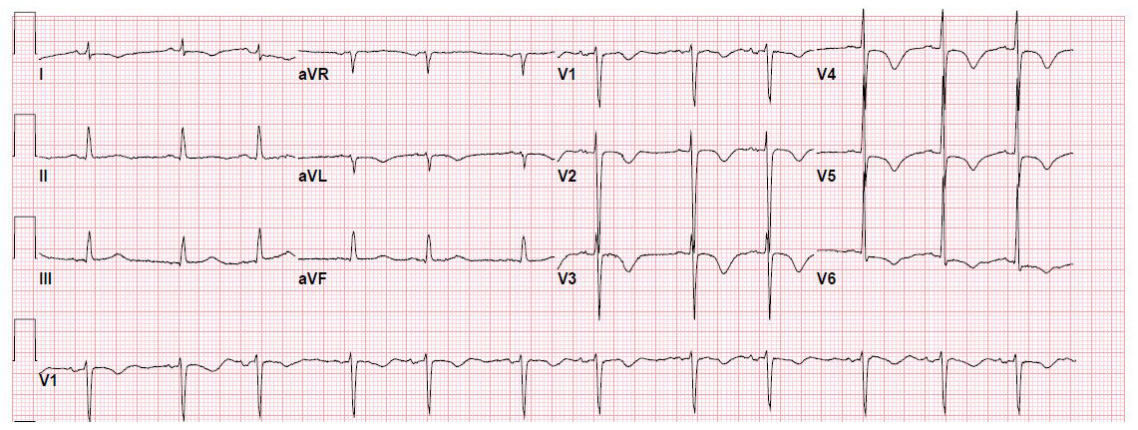
ECG showing symmetrical T-wave inversions in leads V1 through V6 and QTc of 458 milliseconds.

Emergent bedside electrocardiography (ECG) was performed, which revealed a QTc of 458 milliseconds ( Figure 1). ECG also revealed symmetrical T-wave inversions in leads V1 through V6, which was chronic changes related to his underlying coronary disease. Laboratory evaluation revealed, elevated CPK measuring 18000 units/L, Troponin I measuring 7.12 ng/ml. Serum calcium was 13 mg/dL, serum creatinine of 1.6 mg/dL, bicarbonate of 17 millimole/L with the lactate of 4 millimole/L. Metabolic parameters including serum sodium, potassium, magnesium, phosphate, and liver function panels were within normal limits.

Imminent airway compromise with worsening hypertonia was prevented with rapid sequence endotracheal intubation. Considering NMS as the most pertinent differential diagnosis, the patient was treated with intravenous dantrolene sodium at a dose of 1 milligram per kg body weight. Other supportive measures included volume resuscitation with intravenous fluid boluses along with acetaminophen and external cooling blankets for temperature control and intravenous lorazepam for agitation.

After initial stabilization alternative causes of acute change in mental status with generalized hypertonia and hyperthermia were considered. Emergent computed tomography of the brain was negative for acute intracranial bleed, there were no signs of elevated intracranial pressure, midline shift, or hydrocephalus. CNS infection including meningoencephalitis was evaluated with lumbar puncture and cerebrospinal fluid (CSF) analysis. CSF analysis revealed normal protein, glucose, RBCs, and WBCs. CSF was negative for cryptococcal antigen, herpes simplex virus DNA, and west-Nile virus. Brain imaging with MRI could not be performed due to metallic artifacts from a prior bullet injury. The patient did not have a preceding history of seizure before hospitalization and did not have a seizure-like activity after hospitalization. Electroencephalography (EEG) with 24-hour video EEG monitoring was negative for epileptiform activity. As the patient had elevated troponin I, with previous history of CABG, transthoracic echocardiography was performed, which was negative for new regional wall motion abnormality. Thus, elevated troponin was attributed to skeletal muscle sources.

During hospitalization, generalized muscle rigidity and other signs of NMS continued to improve. Anti-dopaminergic agents were avoided and levodopa/carbidopa for parkinsonism was resumed. There was an elevation in serum creatinine consistent with acute kidney injury stage II which was likely secondary to rhabdomyolysis. It was treated conservatively with volume expansion. He did not require renal replacement therapy, and renal function continued to resolve back to baseline. He was extubated on the fourth day of ICU admission. Urinary tract infection, which was his initial admitting diagnosis, was treated with a five-day course of intravenous ceftriaxone based on the pan-sensitive *Escherichia coli* on urine culture. He had an otherwise uneventful clinical recovery except for generalized weakness from deconditioning likely related to skeletal muscle injury from NMS related rhabdomyolysis. He was discharged to a subacute rehabilitation facility for post ICU rehabilitation.

The patient was followed up at the primary care internal medicine clinic and neurology clinic two weeks after hospital discharge. He was noted to have a significant improvement in generalized weakness/deconditioning. Renal function recovered back to baseline. He did not have any worsening of his parkinsonian symptoms including tremor, rigidity, and bradykinesia.

## Discussion

Ziprasidone is a novel atypical antipsychotic drug that acts as an antagonist at human dopamine D2 receptors and serotonin, 5-HT2A, receptors and an agonist at human serotonin, 5-HT1A, receptor, and also selectively inhibits the reuptake of norepinephrine and serotonin in the presynaptic cell ^6^. Clinical studies have shown Ziprasidone as a well-tolerated drug with high efficacy in the treatment of schizophrenia and schizoaffective disorder ^6, 7^. Additionally, the intramuscular use of Ziprasidone has shown good efficacy in the treatment of acute agitation ^6^. It commonly causes adverse effects such as headache, nausea, or drowsiness and rarely been reported to cause extrapyramidal symptoms and QTc prolongation ^7^.

 The systematic review of antipsychotics-induced NMS showed that Ziprasidone-induced NMS occurs abruptly displaying typical symptoms of NMS such as mental status changes, hyperpyrexia, dysautonomia features such as diaphoresis, tachycardia, and fluctuating blood pressure, leukocytosis, and tremor ^4^. The diagnostic criteria set by the DSM-IV includes the presence of hyperpyrexia and severe muscle hypertonia following exposure to neuroleptics and the presence of two or more of the following features: tremor, dysphagia, diaphoresis, altered mental status, incontinence, tachycardia, mutism, labile blood pressure, elevated creatine kinase, and leukocytosis ^8^. Following exposure to repetitive doses of intramuscular Ziprasidone, our patient developed hyperthermia, severe muscle hypertonia, and had tachycardia, tachypnea, diaphoresis, altered mental status, elevated creatine kinase, and elevated troponin, meeting criteria for the diagnosis of the neuroleptic malignant syndrome.

NMS is well recognized with typical antipsychotics; Ziprasidone induced NMS is uncommon and evidence is limited to case-reports ^9– 12^. The classical symptoms such as hyperthermia, muscle rigidity, altered mental status, diaphoresis, tremor, stupor, tachycardia, tachypnea, and diaphoresis, were common in all these cases. The onset of NMS varied among these cases, ranging from abrupt onset to onset after eight weeks of Ziprasidone use. The occurrence of NMS due to antipsychotic drug use is idiosyncratic. The symptoms may appear anytime following exposure to antipsychotic drugs, occurring after exposure to single-dose to occurring after exposure to the same dose of an antipsychotic drug for years ^13^. Despite the diagnostic criteria, Ziprasidone-induced NMS can be diagnostically challenging as it oftentimes presents with atypical clinical features. 

The exact pathophysiologic mechanism behind the development of NMS is unclear, but the blockade in dopamine receptors induced by antipsychotics use is believed to play a crucial role in precipitating the syndrome ^1, 4^. This theory is supported by numerous shreds of evidence, such as NMS has been reported with the use of drugs with dopamine blockade properties or with the withdrawal of dopamine receptor agonists. The hypothalamic dopamine blockade results in hyperthermia and dysautonomia while the nigrostriatal pathway blockade results in extrapyramidal symptoms such as tremors and muscle hypertonia. It is also believed that the muscle hypertonia and the rhabdomyolysis occur from a direct alteration in the skeletal muscle’s mitochondrial function from the use of antipsychotics ^14^. Sympathoadrenal dysfunction is also believed to have contributed to the development of the clinical features of NMS, as an increased adrenergic and serotonergic activity is associated with the NMS and elevated catecholamine levels have been found in NMS cases ^15^. Our patient was showing combative behavior, agitated, and had altered mental status. The possible development of dehydration and physical exhaustion from his combative behavior, which are the risk factors for the development of NMS ^5^, probably had played a role in the precipitation of the NMS following administration of Ziprasidone. Although there isn’t a particular diagnostic test for NMS, and laboratory tests can help confirm the diagnosis, rule out other differentials, and monitor patients for potential complications from NMS. Although NMS is a nearly fatal condition, no fatal or lethal outcome has yet been reported with Ziprasidone-induced NMS ^4^.

It is important to recognize NMS as it is associated with significant morbidity and can occur abruptly after the start of antipsychotic agents such as Ziprasidone. Increasing pieces of evidence advocate the use of antipsychotics such as Ziprasidone for delirium although clinical trials have shown questionable benefits ^16^. So, clinicians should be aware of the potentially life-threatening adverse reaction such as NMS and should remain vigilant to identify NMS anytime, if occurs, throughout the use of conventional antipsychotic drugs and novel agents such as Ziprasidone.

## Conclusion

Neuroleptic malignant syndrome (NMS) is a potentially fatal reaction that can result from the use of atypical antipsychotic agents such as Ziprasidone. NMS is associated with significant morbidity and mortality from rhabdomyolysis, acute renal failure, and sepsis. Increasing body of evidence advocate for the use of Ziprasidone in psychiatric conditions like schizophrenia, bipolar disorder, and delirium, so, clinicians should be aware of the NMS associated with use of Ziprasidone.

## Consent

Written informed consent was obtained from the patient’s son for publication of this case report and accompanying images because the patient was unable to provide consent and the son was next of kin.

## Data availability

All data underlying the results are available as part of the article and no additional source data are required.
