# Supplementary material for: Case Report: Ziprasidone induced neuroleptic malignant syndrome
Source: F1000Res. 2021 Feb 17;10:124. [Version 1] doi: 10.12688/f1000research.51094.1 (PMC7953912; doi:10.12688/f1000research.51094.1)
Supplement: Peer review history

# Peer review: Reviewer response for version 1

**DOI:** 10.5256/f1000research.54209.r79894

**Published:** 2021-03-09

**Authors:** Ashish Saraf

**Affiliations:** 1 Department of Forensic Medicine and Toxicology, All India Institute of Medical Sciences, Gorakhpur, Uttar Pradesh, India

The case report is well written. The authors have given detailed description of the case mentioning the clinical features, the diagnostic workup and treatment given. The other causes of the rigidity have been ruled out during the diagnostic workup. The discussion is also well written and highlighted the importance of this case report. 

 This case report will definitely make the clinicians aware of the fact of NMS in newer drugs and hence making them vigilant.

Are enough details provided of any physical examination and diagnostic tests, treatment given and outcomes?

Yes

Is the case presented with sufficient detail to be useful for other practitioners?

Yes

Is sufficient discussion included of the importance of the findings and their relevance to future understanding of disease processes, diagnosis or treatment?

Yes

Is the background of the case’s history and progression described in sufficient detail?

Yes

Reviewer Expertise:

Forensic medicine, Clinical Forensic Medicine, Forensic Science.

I confirm that I have read this submission and believe that I have an appropriate level of expertise to confirm that it is of an acceptable scientific standard.

# Response

**Published:** 2021-03-11

**Authors:** Alok Atreya

**Affiliations:** Lumbini Medical College, Nepal

Dear Dr. Saraf,

 We thank you for your time to go through our article. We further thank your for highlighting the important findings and positive comments. We are really encouraged.

# Peer review: Reviewer response for version 1

**DOI:** 10.5256/f1000research.54209.r79895

**Published:** 2021-03-05

**Authors:** Prajwal Ghimire

**Affiliations:** 1 Department of Neurosurgery, King's College Hospital NHS Foundation Trust, London, UK

The authors have presented a rare case report of a well recognised drug induced neurologic emergency of Neuroleptic malignant syndrome due to Ziprasidone. Sedhai *et al.* have highlighted major challenges and salient points during management of these conditions including the current knowledge regarding its pathophysiology. The case report raises the awareness regarding this potentially life-threatening condition during use of an emerging drug which is now more commonly used for neuro-psychiatric conditions of schizophrenia and bipolar disorders.

 The case report is well written and highlights the current knowledge and brief literature review in the discussion section with relevant references. It certainly adds a vital information regarding the drug to the current available knowledge in the literature.

Are enough details provided of any physical examination and diagnostic tests, treatment given and outcomes?

Yes

Is the case presented with sufficient detail to be useful for other practitioners?

Yes

Is sufficient discussion included of the importance of the findings and their relevance to future understanding of disease processes, diagnosis or treatment?

Yes

Is the background of the case’s history and progression described in sufficient detail?

Yes

Reviewer Expertise:

Neurological disease, Neurosurgery

I confirm that I have read this submission and believe that I have an appropriate level of expertise to confirm that it is of an acceptable scientific standard.

# Response

**Published:** 2021-03-11

**Authors:** Alok Atreya

**Affiliations:** Lumbini Medical College, Nepal

Dear Dr. Ghimire,

 We appreciate your time you took to go through our article and write such a detailed review. We would further thank you for your positive and encouraging comments on our article.
